# The Efficacy of the Addition of tDCS and TENS to an Education and Exercise Program in Subjects with Knee Osteoarthritis: A Randomized Controlled Trial

**DOI:** 10.3390/biomedicines12061186

**Published:** 2024-05-27

**Authors:** Joaquina Montilla-Herrador, Jose Lozano-Meca, Juan Vicente Lozano-Guadalajara, Mariano Gacto-Sánchez

**Affiliations:** 1 Department of Physical Therapy, Faculty of Medicine, CEIR Campus Mare Nostrum, University of Murcia, Instituto de Investigación Biosanitaria-Virgen de la Arrixaca (IMIB), El Palmar, 30120 Murcia, Spain; montilla@um.es (J.M.-H.); marianogacto@um.es (M.G.-S.); 2 Physical Medicine and Rehabilitation Service, Morales Meseguer University Hospital, 30008 Murcia, Spain; juanv.lozano@carm.es

**Keywords:** knee osteoarthritis, chronic pain, exercise, transcranial direct current stimulation, TENS

## Abstract

Knee osteoarthritis (KOA) has a significant impact on patients’ quality of life. This study aimed to assess the effectiveness of integrating transcranial direct current stimulation (tDCS) and transcutaneous electrical nerve stimulation (TENS) into an education and exercise program with the aim of decreasing pain and improving physical function in KOA. A randomized controlled trial with 65 KOA patients was conducted. The subjects were assigned to one of the following three groups: education and active exercise plus (1) double active tDCS and TENS, (2) active tDCS and sham TENS, and (3) double sham tDCS and TENS. Sessions were conducted over a 20 min period, whilst data on pain, chronic pain clinical variables, and physical function were collected. Although all groups showed improvement in pain-related symptoms in the short and medium term, the addition of tDCS and/or TENS did not significantly enhance the benefits of the exercise and education program. These findings suggest that an education and active exercise program in the treatment of KOA has a positive effect on pain, with or without the addition of tDCS and/or TENS.

## 1. Introduction

Knee osteoarthritis (KOA) is a musculoskeletal joint pathology of multifactorial origin that presents a progressive degenerative component involving all joint tissues (cartilage, subchondral bone, synovial membrane, menisci, and infrapatellar fat pad) [1]. The most common symptoms are persistent pain and decline in physical performance, especially at advanced ages [2]; these symptoms in KOA, alongside its high prevalence, have the potential to saddle the global health infrastructure with an important socioeconomic burden [3]. In patients with KOA, socioeconomic and educational level, as well as psychosocial factors (such as cognitive ability and the ability to manage anxiety; factors linked, in turn, to kinesiophobia or catastrophizing, among others), have an impact on the prevalence of disability and chronic pain symptoms [4,5]. The development of low-cost, non-surgical and non-pharmacological treatments to improve patient outcomes has been identified as a key priority area for people living with KOA [6].

KOA is not necessarily associated with pain and, likewise, not all the pain KOA patients experience is a result of KOA itself. An estimated prevalence of asymptomatic KOA ranging from 19% to 43% has been observed by magnetic resonance imaging (MRI) in older adults [7]; hence, the presence of symptoms such as pain and limitation in physical performance may not be totally due to the presence of KOA. Indeed, the alteration in the central mechanisms, or the patient perception and interpretation of pain, may also play a key-role [7]. Previous studies suggest a potential alteration in the central processing of persistent pain secondary to changes due to KOA, like infrapatellar or synovial inflammation [2], generating, in turn, changes in the primary motor and dorsolateral prefrontal cortex that imply a decrease in descending pain inhibition mechanisms and a central sensitization [2,8,9].

Active exercise (aerobic or strengthening) is the first-choice conservative treatment for KOA and it is recommended in all international clinical practice guidelines [10,11]. Although exercise is effective in KOA, some studies highlight that the benefits of this treatment are moderate for pain and physical function in the short term, and these studies extoll patient education as a cornerstone of the therapeutic approach [11].

Other therapeutical strategies include transcranial direct current stimulation (tDCS), and transcutaneous electrical nerve stimulation (TENS) [12]. The first approach, tDCS, is a non-invasive brain stimulation technique with the potential to enhance brain connectivity, subsequently improving pain perception in subjects with chronic pain [13]. Its operational principles consist of applying weak direct currents through electrodes placed in the scalp and acting on brain regions beneath the electrode, as well as stimulating interconnected distal regions [14]. The results of studies carried out on subjects with chronic pain suggest that tDCS used in the primary motor cortex (M_1_) can reduce pain and improve the psychosocial aspects associated with pain, by modulating pain processing in both cortical and subcortical regions, facilitating descending antinociceptive pathways and inducing neuroplastic changes in the underlying areas of the brain regions [15,16]. TENS is widely used in the management of chronic pain to relieve pain and facilitate activity performance [17].

In the specific field of KOA, TENS has been shown to improve pain, gait, and functional physical capacity in subjects with moderate KOA [17,18]. When combining TENS with a strengthening exercise program, higher levels of improvement have been observed with respect to the isolated implementation of the exercise program [19]. Both therapeutic strategies (tDCS and TENS) have a potential impact on pain and dysfunction, but not on specific joint-related aspects (e.g., stiffness) [13,18]. Previous studies have also shown that the combination of tDCS with TENS in subjects with chronic pain displayed higher levels of improvement than with the application of tDCS alone [20,21]. When focusing on the specific application of tDCS, an increased cortical excitability has been found on KOA patients [22]. Also, different psychosocial factors (such as depression or the self-perceived mental component of health-related quality of life) have been defined as predictors for the analgesic response to tDCS [23]. Many meta-analyses focusing on tDCS have been performed on different painful conditions to explore its effects on pain [24,25]. In the specific field of KOA, two meta-analyses recently examined the effect of tDCS on pain [26,27] but neither functional performance nor the psychosocial components of pain (kinesiophobia, catastrophizing), inherent in chronic pathologies, were explored.

The primary objective of the present study was therefore to determine the effectiveness of the addition of tDCS and/or TENS to an education and exercise program on the levels of pain in the short, medium, and long term in KOA patients. The secondary objectives were to evaluate the effect of adding tDCS and/or TENS to an education and exercise program on physical function, central sensitization, and certain psychosocial components of pain: uncertainty, catastrophizing, kinesiophobia and quality of life.

## 2. Materials and Methods

### 2.1. Study Design

The study corresponds to a three-arm, single-blinded controlled randomized clinical trial. The study was conducted according to the standards of the Consolidated Standards of Reporting Trials CONSORT recommendations [28]. The trial was previously registered in clinicaltrials.gov (NCT05138471). This study was submitted to and approved by the Ethics Committees of both the University of Murcia (3536/2021) and the Morales Meseguer University Hospital (ENS 14/21), and it was conducted with patients from the Morales Meseguer University Hospital (Murcia, Spain). The study protocol for this trial was previously published [29].

### 2.2. Eligibility Criteria

#### 2.2.1. Inclusion Criteria

Adults with a radiographical diagnosis of KOA with Kellgren–Lawrence Classification of grade I, II, III or IV [30]; over fifty years of age, chronic pain for more than six months and compliance with the diagnostic criteria for KOA from the guidelines by the Osteoarthritis Research Society International (OARSI) [31], i.e., pain on visual analogue scale (VAS) of over 4/10, presence of morning stiffness below 30 min, absence of hyperthermia upon palpation in the joint, presence of alteration of the bone image, and presence of crepitation under gravity.

#### 2.2.2. Exclusion Criteria

Patients were excluded if they met any of the following exclusion criteria:

- Patients sensitive to providing biased and/or low-quality information, discriminated through the Mini-Mental State Examination [32,33].
- Severe hearing, visual or sensory impairments, or significant comorbidities preventing the adequate performance of physical activities and questionnaires.
- Patients undergoing knee replacement surgery on the contralateral knee at any time.
- Patients having received invasive intervention for pain (infiltration, blocks, etc.) in the 6 weeks before the study onset.
- Pain due to musculoskeletal conditions such as acute tendinopathies or meniscopathies, knee fracture, and low back pain with sciatica or fibromyalgia.
- Patients who declined to participate out of fear or denial of therapy.
- Severe heart disease preventing exercise.
- Body mass index above 45.
- Patients with neuropsychiatric disorders (schizophrenia, epilepsy, or bipolar disorders) or under medication for these conditions.
- Patients with metallic implants in the area (skull or knee).

### 2.3. Participant Recruitment and Randomization

Study subjects were recruited from the Rehabilitation, Traumatology and Rheumatology departments of the Morales Meseguer University Hospital (Murcia, Spain). Participants were informed about the different treatments available for their condition in case they did not accept therapy. Subjects were contacted by telephone to assess eligibility criteria and, if applicable, to proceed to the first evaluation. An information sheet was initially provided, and the corresponding informed consent was signed.

**Figure 1:**
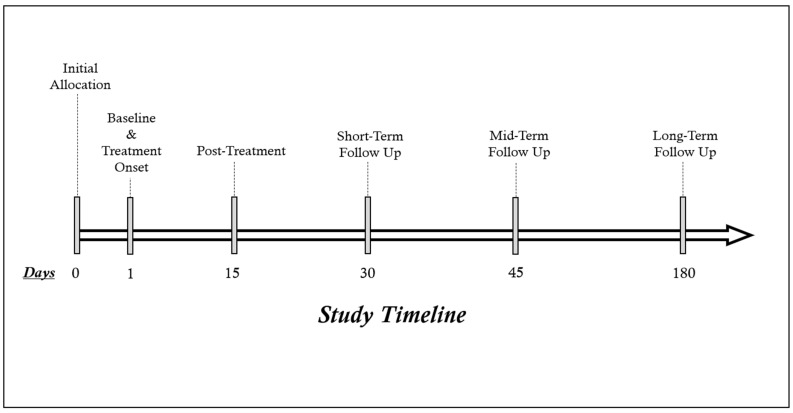
Assessment timeline of the study.

Sociodemographic and clinical variables were obtained through self-administered and hetero-administered questionnaires during the first interview. The baseline [day (D)1], second (post-treatment) [D15] and fourth (monthly) [D30] evaluation were carried out on site, in person, whilst the third [D45] and fifth [D180] evaluations were carried out by telephone two weeks and six months after finishing the treatment, respectively. The intervention was carried out in consultation by one of the researchers. The phone-call from the third assessment was also used to increase adherence to the intervention plan by providing subjects with a brief reminder about the benefits of adhering to the intervention patterns. The patients were blinded and did not know the treatment they were receiving. Further details on the timeline of the research are displayed in Figure 1.

Concerning group randomization, the investigator conducting the therapy opened randomly arranged and consecutively numbered opaque envelopes, organized by a second assistant (MGS) and containing the group assignment after enrollment in a 1:1:1 ratio.

### 2.4. Outcome Measures

#### 2.4.1. Sociodemographic and Clinical Variables

Sociodemographic and clinical variables were collected to characterize the sample, such as gender, age, weight, height, BMI, employment status, level of education, pharmacology used and clinical variables such as duration of symptoms, the osteoarthritic knee affected, the degree of Kellgren/Lawrence (K/L) involvement according to the four grade classification [30], presence of walking assistance devices and level of physical activity using the International Physical Activity Questionnaire (IPAQ) [34].

#### 2.4.2. Pain and Symptoms of KOA

Visual analog scale (VAS) was used to assess patient’s pain [35]. In order to evaluate the symptoms of KOA (pain, stiffness, and functionality), the Spanish version of the Western Ontario and McMaster Universities Osteoarthritis Index (WOMAC) scale was used [36].

#### 2.4.3. Psychosocial Components of KOA

Catastrophizing was measured using the Pain Catastrophizing Scale (PCS) [37], whilst the Spanish version of the Tampa Scale of Kinesiophobia (TSK) [38,39] was used to assess kinesiophobia. The Spanish version of the Mishel Uncertainty Scale (MUIS) assessed the uncertainty related to KOA [40]. With the aim of measuring the somatic and emotional symptoms commonly associated with the central sensitization syndrome, we used the Spanish adaptation of the Central Sensitization Inventory (CSI) [41]. For anxiety and depression we used the Hospital Anxiety and Depression Scale (HAD) [42,43]. Finally, the Spanish version of the European Quality of Life–5 Dimensions (EuroQol-5D) was used to assess quality of life [44].

#### 2.4.4. Physical Function

Mobility was evaluated through two walking performance tests: the 6-min walk test (6MWT), and the 10 m walk test (10mWT), widely used in the field of KOA [45]. As for balance and strength, the Short Physical Performance Battery (SPPB) was used [46]. Quadriceps strength was measured with handheld dynamometer (MicroFET2—Force Evaluation and Testing. Hoggan Scientific LLC., Salt Lake City, UT, USA), following the protocol displayed by Bohannon [47]. Finally, the Timed Up and Go Test (TUGT) was used to assess patients’ functional mobility [48].

### 2.5. Procedure

The therapy consisted of applying tDCS and/or TENS in addition to an active exercise and education program. The intervention consisted of five sessions on alternate days, for two weeks overall. The therapy was applied in three defined groups, as follows: Group 1 received active tDCS and active TENS; Group 2 received active tDCS and sham TENS; Group 3 received sham tDCS and sham TENS.

#### 2.5.1. Transcranial Direct Current Stimulation (tDCS)

Active tDCS therapy was administered through a portable device (HDC-Stim, Newronika, Milan, Italy) with two 35 cm^2^ electrodes imbibed with saline solution. The anode was placed over the M_1_ cortex contralateral to the treated knee, and the cathode laid on the ipsilateral supraorbital (SO) area [49]. An intensity of 1.5 mA was applied, with the aim of avoiding potential side effects due to a higher intensity. For the sham application, current increased from 0 to 1.5 mA for 15 s (generating an initial sensation of itching), and then decreased to 0 mA, subsequently remaining with no stimulation until the end of the session [50]. The total length of the session corresponded to 20 min.

#### 2.5.2. Transcutaneous Electrical Nerve Stimulation (TENS)

The active application of TENS (Dual Channel TENS Model 120Z, ITO Co., Ltd., Tokyo, Japan) consisted of placing two 5 × 5 cm^2^ electrodes on both sides of the kneecap of the affected knee, with stimulation parameters of 250 Hz, and 60 ms pulse width. Intensity gradually increased until the patient indicated a perception below the visible motor threshold, as well as a non-painful sensation, for 20 min. Concerning the application, patients were previously informed that they could eventually feel nothing, owing to the expected effect being below the sensory threshold [51]. This strategy was adopted to minimize the risk of masking-related bias.

#### 2.5.3. Exercise and Education Program

A basic exercise program was applied to strengthen the knee and hip of the affected limb through the NE-MEX (neuromuscular exercise) exercise program for KOA [52,53]. The training sessions consisted of three different parts: (i) warm-up, (ii) circuit program, and (iii) cool-down. The circuit program included four exercise circles with the aim of targeting core stability, postural orientation, muscle strength and functional exercises. Each exercise was performed according to the following pattern: 2–3 sets of 10–15 repetitions, with rest between each set and exercise. Three gradual levels of difficulty were set for each exercise and progressions were defined by the physical therapist, based on the evolution of each patient’s performance. The program was initially explained and applied on-site, during the first consultation, and its implementation and compliance was performed at home and throughout the consecutive sessions. The pain educational program was carried out by teaching and focusing on “non-avoidance” behaviors on the presence of mild knee pain, self-management tips to cope with exercise loads, and watching a video on central sensitization of pain created by the Spanish Society of Pain (available online: www.youtube.com/watch?v=JYA_mrNuLz0, accessed on 2 May 2024).

### 2.6. Statistical Analyses

Sample size was calculated considering the study by Chang et al. [21] in which a similar therapeutic approach was adopted among subjects with KOA. Since their post-test results on our main outcome variable (pain) displayed the mean values (95% confidence intervals, 95% CI) of 24.1 (33.4, 14.8) and 33.7 (49.0, 18.5), respectively, the standard error of the mean (SEM) was calculated, and the subsequent standard deviations were obtained [54]. The effect size of the intervention was therefore quantified (Cohen’s d = 0.56) and used as the anticipated effect of the intervention. Adopting an alpha level of 0.05 (95% CI), an anticipated power (1-β) of 0.80 and considering three treatment groups (according to our previously described design), the minimal sample size needed corresponded to 36 subjects. Sample size calculation was performed by means of G*Power version 3.1.9.7 (G*Power, Heinrich-Heine-Universität Düsseldorf, Düsseldorf, Germany).

Data distribution was previously tested for normality through Shapiro–Wilk (n ≤ 50) or Kolmogorov–Smirnov (n > 50) tests, when applicable. *t*-tests (or Mann–Whitney U-tests, when applicable) were used to assess potential between-group comparisons of baseline characteristics for continuous variables, whilst chi-squared tests were applied to assess differences across categorical variables.

Concerning between-group comparisons in the different timepoints measured, ANOVA tests were used (if necessary, the Kruskal–Wallis test was used as the non-parametric equivalent to ANOVA). In the eventuality of statistical differences, effect sizes would be calculated to assess the magnitude of the effect. As for the intragroup changes through time, ANOVA for repeated measures (or its equivalent non-parametric test, Friedman test) was used and, if necessary, Tukey post hoc (or Dunn–Bonferroni, for non-parametric) analyses were performed. For the analyses of pain, as the main outcome measure, in the mid/long term (T3 = day 30; T4 = day 45; T5 = day 180), missing data were not replaced, but the tendency of the data lost through time was analyzed through specific multiple imputation for handling missing data, following the patterns and guidelines previously defined [55,56], and one-factor ANOVA was performed to assess potential statistically significant differences across groups after imputation.

Results are presented, unless otherwise stated, as follows: for binary and/or categorical variables, counts and percentages; for continuous variables, means and standard deviation. All analyses were performed using IBM SPSS Statistics for Windows, Version 28.0 (IBM Corp.: Armonk, NY, USA; 2021), with a p-level of significance set at *p* < 0.05.

## 3. Results

**Figure 2:**
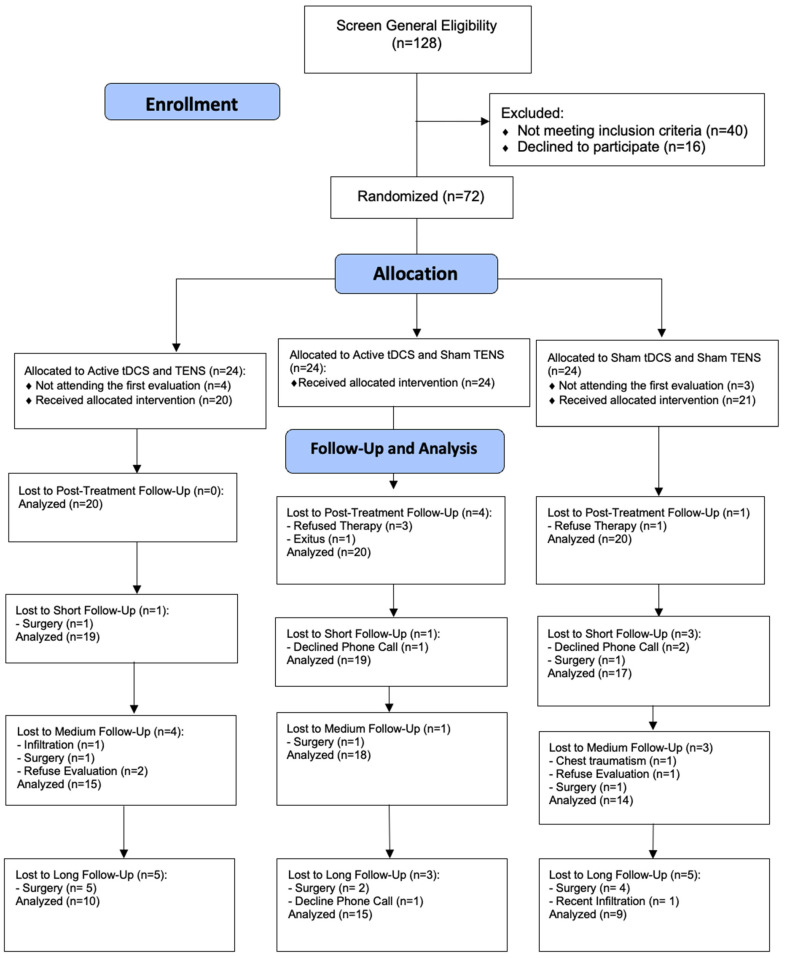
Flowchart of patient enrollment.

A total of 128 potential participants were initially screened for eligibility: amongst them, 72 subjects met inclusion criteria and they were subsequently allocated to each group. After allocation, 65 subjects (53.7%) completed baseline assessment (20 (30.8%) in Group 1; 24 (36.9%) in Group 2; and 21 (32.3%) in Group 3). At treatment onset, four subjects refused the intervention (after having been randomized and allocated), and one subject was deceased. Finally, a total of 60 participants completed treatment and post-intervention timepoints. Further assessment details of the enrollment and allocation process are described in Figure 2.

The participants’ mean age was 68.05 ± 8.65 years of age. Around 70% of the sample corresponded to women, and 60% of the study subjects were professionally retired. The sociodemographic characteristics of all participants at baseline were balanced across the groups, as displayed in Table 1.

Table 2, Table 3 and Table 4 display the results for pain, psychosocial components of pain (uncertainty, catastrophizing, kinesiophobia and quality of life), and physical function, respectively. All groups experienced improvement in their levels of pain in the short-term; this effect waned over time. There were no differences in the level of pain across treatment groups at any time-point measured. Concerning psychosocial components of pain, the change in scores did not reach statistical significance across most of the variables. No significant differences were stated concerning each of the treatment groups whatsoever across any time-period during the study. Focusing on the physical function dimension, measures showed a numerical tendency towards improvement, not reaching statistical significance either. All groups experienced a similar pattern, with no differences across treatment groups in the different time periods assessed.

The analyses of within-group changes through time on pain revealed a general pattern of significant changes from baseline to each one of the subsequent time-points measures, while the general tendency for the variables focusing on psychosocial components of pain and physical function showed scattered statistically significant changes through time, mainly between baseline and day 45.

Multiple imputation on pain-related variables led to datasets of imputed data after five iterations, which were analyzed through one-factor ANOVA tests: the statistical significance remained over 0.05 in all cases, thereby stating the absence of differences across the three treatment groups, as displayed in Table 5.

As for the eventual appearance of adverse events, itching was reported by 13 (20%) patients and 20 (30.8%) reported stinging in the electrode area, 48 (73.8%) patients showed redness of the skin related to the stimulation area, 6 (9.2%) patients suffered from headache after stimulation, only 1 (1.5%) patient reported sleeplessness. No serious adverse effects were reported.

## 4. Discussion

To the best of our knowledge, this is the first study to focus on the addition of tDCS and/or TENS to an education and active exercise treatment, taking into account the short-, medium- and long-term effects, as well as both the physical and sociopsychological variables of chronic knee pain. The current study aimed to assess the impact on the therapeutic effects from the potential addition of tDCS and/or TENS to an education and active exercise program in the treatment of KOA on pain, as the main objective. Since no differences exist across the three treatment groups, the first conclusion is therefore that the application of an educational and active exercise program has a positive effect on pain, but there is no evidence to support a potential differential effect across the three modalities of a therapeutic program in addition to the exercise program (i.e., either alone, with tDCS, or with tDCS and TENS). The secondary objectives were to evaluate the effect of adding tDCS and/or TENS to an education and exercise program on physical function and the psychosocial components of pain (uncertainty, catastrophizing, kinesiophobia, and quality of life); again, the application of the education and active exercise program is positive in its own, regardless of the addition of tDCS, TENS, or tDCS and TENS. No differences were found in the aforementioned variables across treatment groups at any timepoint measured, a fact that endorses that there are not differential effects linked to the addition of any of the aforementioned components (tDCS or TENS).

An overall comparison with other trials covering all our dimensions is therefore not possible, but several previous studies have approached the analytic comparison of specific components of the therapeutic approaches applied in our study. Previous studies have explored the effects of active tDCS versus sham tDCS in KOA [20,57] and showed improvements in self-reported pain in the short- and medium-term, but no other therapies were applied in combination with tDCS. Also, the application of tDCS and TENS either in combination or individually produced differences in pain, but no differences in terms of physical function (measured through the 6MWT) were reported [58].

Chang et al. (2017) [21] found similar levels of improvement in pain and function when applying active tDCS and strengthening versus sham tDCS and strengthening. These findings, along with the results stemming from our study, reinforce the key-role of education and active exercise as the cornerstone components of the therapeutic approach of KOA, as already suggested by previous authors: Naugle et al. (2017) [59] observed and described the relationship between physical activity and the endogenous pain inhibitory system associated with less perceived pain [59]. Moreover, greater benefits are stated when applying the NEMEX protocol combined with exercise education for 12 weeks to reduce pain intensity [60]. In line with our results, Sajadi et al. (2020) [61] also examined the addition of tDCS and TENS to an active strengthening exercise program, stating no differences in pain- and function-related outcomes, reaffirming the hypothesis that exercise plays a key role in clinical improvement across groups. The possible “void effects” of the addition of tDCS have also been observed in pathologies differing from KOA, as in musculoskeletal conditions and stroke, among others [62,63].

The results from the current study should be interpreted in the light of its limitations. Firstly, the different follow-up timepoints displayed a consecutive loss of subjects, mainly due to the fact that some participants were destined for surgery for KOA; we tried to compensate for this limitation through the multiple imputation analyses carried out, leading to similar values of non-significance. Secondly, even though baseline scores were adequately balanced for all the main outcome variables explored in the current study, the specific measurement of strength in the left affected leg (n = 33; *p*-value = 0.023) revealed differences in baseline distribution across groups. This variable was, however, not a “core-measure” in our study and, moreover, the need to provide balanced variables across groups in baseline measurement is not only not recommended but potentially misleading [64,65]. Moreover, it is not included in the CONSORT checklist since, if randomization is adequately performed, the presence of potential baseline differences does not constitute a bias. Third, the therapeutic procedure of the tDCS used was based on the application of 1.5 mA; since some other studies in the field use 2.0 mA, this fact could have potentially hindered better results in the groups in which active tDCS was implemented. Notwithstanding the aforementioned, similar studies in the field have worked with intensities of 1 mA [21], in line with the findings by Brunoni et al. (2012) [66], supporting the idea of applying currents in a 1–2 mA range. Finally, the current research was based on a three-arm design. The implementation of a factorial clinical trial under the design A0 vs. B0 vs. AB vs. 00 (with “A” being tDCS, “B” being TENS, and “0” being the corresponding sham application) could have been clinically relevant. The decision was adopted based on (i) the sample anticipated (the partition into four different groups instead of three would have lowered the study power) and (ii) the intention to explore the effects of tDCS on KOA as one of the main focuses of the research. However, since neither tDCS alone nor in combination with TENS showed a significant difference across the variables from exercise alone, which of these additional therapies is driving this difference is, therefore, less relevant.

Future research should develop further standardization parameters of stimulation and tDCS-inherent device parameters, since, to date, different tDCS devices can be found throughout neuromodulation laboratories worldwide with characteristics differing widely; several factors therefore need to be defined [66]. Also, implementing a complete pain neuroscience education program and/or specific biobehavioral-based therapeutic education program aside from the active exercise and education program may be a relevant strategy in order to provide the patient with self-empowerment to cope with pain [67,68]. Moreover, registering the level of adherence and daily performance of the active exercise component may provide a more specific picture leading to the implementation of tailored interventions in the treatment and therapeutic management of KOA.

## 5. Conclusions

No statistical differences existed with respect to pain, the psychosocial components of pain, or physical function when adding either tDCS and/or TENS to the program, neither immediately after treatment completion, nor after six months. An education and active exercise program for the treatment of KOA provides positive effects on pain, with or without the addition of tDCS and/or TENS. Therefore, the findings from the current research do not support or endorse the additive effect of tDCS and/or TENS to an educational and active exercise program for adults with pain linked to KOA.

## Figures and Tables

**Table 1:** Baseline sociodemographic and clinical characteristics (n = 65).

Variables		Mean ± SD or N (%)				*p* Value
Sociodemographic Variables		Total	Group 1 (n = 20)	Group 2 (n = 24)	Group 3 (n = 21)	
Age (years)		68.05 ± 8.65	68.70 ± 9.77	64.96 ± 7.20	70.95 ± 8.26	0.060
Gender						
	Male	18 (27.7)	6 (30.0)	5 (20.8)	7 (33.3)	0.622
	Female	47 (72.3)	14 (70.0)	19 (79.2)	14 (66.7)	
Weight (kg)		77.44 ± 13.06	77.83 ± 13.90	76.46 ± 14.47	78.19 ± 10.94	0.897
Height (cm) BMI		163.37 ± 7.55 28.90 ± 3.58	163.55 ± 7.33 28.98 ± 3.94	163.50 ± 8.10 28.45 ± 3.82	163.05 ± 7.40 29.34 ± 3.01	0.973 0.707
Labor situation						
	Active	17 (26.2)	4 (22.0)	10 (41.7)	3 (14.3)	0.066
	Sick leave	5 (7.7)	1 (5.0)	4 (16.7)	-	
	Housewife/ Househusband	4 (6.2)	1 (5.0)	1 (4.2)	2 (9.5)	
	Retired	39 (60.0)	14 (70.0)	9 (37.5)	16 (76.2)	
Level of Studies						
	No formal education	5 (7.7)	2 (10.0)	-	3 (14.3)	0.230
	Primary studies	26 (40.0)	9 (45.0)	8 (33.3)	9 (42.9)	
	Secondary studies	14 (21.5)	3 (15.0)	5 (20.8)	6 (28.6)	
	Higher education	20 (30.8)	6 (30.0)	11 (45.8)	3 (14.3)	
Pharmacology						
	No	2 (3.1)	1 (5.0)	-	1 (4.8)	0.748
	General drugs	13 (20.0)	5 (25.5)	3 (12.5)	5 (23.8)	
	Pain drugs	11 (16.9)	2 (10.0)	5 (20.8)	4 (19.0)	
	Both (general + pain drugs)	39 (60.0)	12 (60.0)	16 (66.7)	11 (52.4)	
Clinical Variables						
Duration of symptoms (months) Right knee affected Left knee affected Both knees affected (right predominance) Both knees affected (left predominance)		51.83 ± 43.69 19 (29.2) 14 (21.5) 13 (20.0) 19 (29.2)	62.40 ± 56.23 5 (25.0) 5 (25.0) 5 (25.0) 5 (25.0)	44.54 ± 33.13 5 (20.8) 5 (20.8) 6 (25.0) 8 (33.3)	50.10 ± 40.80 9 (42.9) 4 (19.0) 2 (9.5) 6 (28.6)	0.399 0.652
Kellgren–Lawrence degree						
	I	1 (1.5)	-	1 (4.2)	-	0.648
	II	23 (35.4)	7 (35.0)	9 (37.5)	7 (33.3)	
	III	38 (58.5)	11 (55.0)	14 (58.3)	13 (61.9)	
	IV	3 (4.6)	2 (10.0)	-	1 (4.8)	
Pain Visual Analogue Scale		6.66 ± 1.55	6.85 ± 1.42	6.50 ± 1.38	6.67 ± 1.88	0.764
Assistive devices for walking						
	No	55 (84.6)	16 (80.0)	21 (87.5)	18 (85.7)	0.779
	Yes	10 (15.4)	4 (20.0)	3 (12.5)	3 (14.3)	
HAD Scale		10.90 ± 7.46	13.78 ± 10.06	9.87 ± 4.72	9.47 ± 6.89	0.131
IPAQ						
	Low	31 (47.7)	10 (50.0)	12 (50.0)	9 (42.8)	0.747
	Moderate	31 (47.7)	9 (45.0)	12 (50.0)	10 (47.6)	
	High	3 (4.6)	1 (5.0)	-	2 (9.5)	
WOMAC		41.36 ± 15.31	46.40 ± 18.14	40.75 ± 11.29	37.28 ± 15.73	0.159
WOMAC (pain)		8.26 ± 3.74	9.00 ± 4.09	8.58 ± 3.34	7.19 ± 3.77	0.267
WOMAC (stiffness)		3.21 ± 1.69	3.60 ± 1.66	3.37 ± 1.52	2.66 ± 1.82	0.179
WOMAC (function)		29.89 ± 11.80	33.80 ± 13.52	28.79 ± 9.07	27.42 ± 12.40	0.192
PCS		15.70 ± 12.16	18.00 ± 14.30	16.33 ± 12.04	12.80 ± 9.90	0.380
TSK-11		30.46 ± 7.37	30.90 ± 6.58	29.58 ± 7.75	31.04 ± 7.88	0.768
MUIS		49.55 ± 10.78	48.95 ± 12.37	50.00 ± 9.20	49.61 ± 11.34	0.951
CSI		32.53 ± 15.73	35.15 ± 20.91	35.20 ± 13.47	27.90 ± 11.45	0.155
EQOL-5D		6.64 ± 3.31	7.65 ± 3.89	6.08 ± 2.88	6.33 ± 3.10	0.261
SPPB		7.15 ± 2.29	7.20 ± 2.54	7.08 ± 2.18	7.19 ± 2.29	0.983
TUGT						
	<10 s	7 (10.8)	2 (10.0)	3 (12.5)	2 (9.5)	0.964
	10–20 s	47 (72.3)	14 (70.0)	18 (75.0)	15 (71.4)	
	>20 s	11 (16.9)	4 (20.0)	3 (12.5)	4 (19.0)	
6-min walk test (m)		238.92 ± 56.59	238.84 ± 66.33	242.46 ± 56.01	234.95 ± 49.87	0.909
10 m walk test (s)		18.31 ± 5.55	18.58 ± 6.43	17.65 ± 4.92	18.80 ± 5.54	0.763
Dynamometer affected right leg (n = 32)		11.04 ± 3.77	12.31 ± 3.68	11.37 ± 3.61	9.55 ± 3.85	0.240
Dynamometer affected left leg (n = 33)		11.42 ± 3.87	11.52 ± 2.12	9.49 ± 2.63	13.84 ± 5.26	0.023 *

Abbreviations: SD, standard deviation; N, number; BMI, body mass index; HAD, Hospital Anxiety and Depression Scale; IPAQ, International Physical Activity Questionnaire; WOMAC, Western Ontario McMaster Universities Osteoarthritis Index; PCS, Pain Catastrophizing Scale; TSK, Tampa Scale of Kinesiophobia; MUIS, Mishel Uncertainty in Illness Scale; CSI, Central Sensitization Index; EQOL, Euro Quality of Life; SPPB; Short Physical Performance Battery seconds; TUGT, timed up and go test; m, meters; s, seconds. * *p* < 0.05.

**Table 2:** Treatment effects on pain at specified time points (days from baseline). All values are means and standard deviation.

Outcome		T1 (D1) G1 = 20 G2 = 24 G3 = 21	T2 (D15) G1 = 20 G2 = 20 G3 = 20	T3 (D30) G1 = 19 G2 = 19 G3 = 17	T4 (D45) G1 = 15 G2 = 18 G3 = 14	T5 (D180) G1 = 10 G2 = 15 G3 = 9	*p*-Value
VAS							
	G1 G2 G3 *p*-value	6.85 ± 1.42 6.50 ± 1.38 6.67 ± 1.88 0.764	4.25 ± 2.02 4.35 ± 2.34 4.65 ± 2.34 0.842	3.74 ± 1.75 3.84 ± 2.45 4.12 ± 2.05 0.858	4.80 ± 2.42 4.56 ± 2.81 4.43 ± 2.50 0.926	5.60 ± 2.22 4.40 ± 2.99 5.78 ± 2.53 0.388	<0.001 *^abc^ 0.004 *^abc^ 0.004 *^abc^
WOMAC (total)							
	G1 G2 G3 *p*-value	46.40 ± 18.14 40.75 ± 11.29 37.28 ± 15.73 0.159	29.00 ± 15.81 29.15 ± 14.40 29.00 ± 12.93 0.999	22.89 ± 12.23 24.05 ± 11.59 21.82 ± 11.17 0.850	31.60 ± 17.75 28.44 ± 14.08 32.50 ± 17.98 0.762	23.10 ± 11.31 21.33 ± 14.99 27.88 ± 16.15 0.558	<0.001 *^abcd^ <0.001 *^abcd^ 0.033 *^b^
WOMAC (pain)							
	G1 G2 G3 *p*-value	9.00 ± 4.09 8.58 ± 3.34 7.19 ± 3.77 0.267	5.40 ± 3.01 5.45 ± 3.30 5.45 ± 2.37 0.998	4.52 ± 2.63 4.36 ± 2.62 4.11 ± 2.11 0.885	5.33 ± 2.47 6.05 ± 3.43 6.92 ± 4.54 0.499	4.60 ± 1.95 4.26 ± 3.15 6.55 ± 4.15 0.226	<0.001 *^abcd^ <0.001 *^abd^ 0.056
WOMAC (stiffness)							
	G1 G2 G3 *p*-value	3.60 ± 1.66 3.37 ± 1.52 2.66 ± 1.82 0.179	1.80 ± 1.19 2.75 ± 1.91 2.20 ± 1.50 0.166	1.42 ± 1.30 1.42 ± 1.46 1.11 ± 1.16 0.736	2.40 ± 1.80 2.22 ± 1.55 2.07 ± 1.49 0.862	1.90 ± 1.85 2.06 ± 1.75 1.77 ± 1.78 0.926	<0.001 *^abd^ 0.004 *^b^ 0.055 *^b^
WOMAC (function)							
	G1 G2 G3 *p*-value	33.80 ± 13.52 28.79 ± 9.07 27.42 ± 12.40 0.192	21.80 ± 12.44 20.95 ± 9.91 21.35 ± 10.68 0.971	16.94 ± 9.54 18.26 ± 9.22 16.58 ± 9.08 0.848	23.86 ± 14.41 20.16 ± 10.40 23.50 ± 13.27 0.651	16.70 ± 8.56 15.00 ± 10.65 19.77 ± 10.80 0.541	<0.001 *^abd^ <0.001 *^bcd^ 0.064 *^b^

* *p* < 0.05; ^a^ post hoc: difference between baseline and day 15; ^b^ post hoc: difference between baseline and day 30; ^c^ post hoc: difference between baseline and day 45; ^d^ post hoc: difference between baseline and day 180. Abbreviations: VAS, visual analogue scale; WOMAC, Western Ontario McMaster Universities Osteoarthritis Index; D, day.

**Table 3:** Treatment effects on psychosocial components of pain at specified time points (days from baseline). All values are means and standard deviation.

Outcome		T1 (D1) G1 = 20 G2 = 24 G3 = 21	T2 (D15) G1 = 20 G2 = 20 G3 = 20	T4 (D45) G1 = 15 G2 = 18 G3 = 14	*p*-Value
PCS					
	G1 G2 G3 *p*-value	18.00 ± 14.30 16.33 ± 12.04 12.80 ± 9.90 0.380	14.20 ± 14.48 13.85 ± 9.99 10.55 ± 9.45 0.547	9.93 ± 10.40 13.66 ± 10.35 8.42 ± 8.32 0.302	0.222 0.667 0.398
TSK-11					
	G1 G2 G3 *p*-value	30.90 ± 6.58 29.58 ± 7.75 31.04 ± 7.88 0.768	26.15 ± 7.87 30.35 ± 5.83 27.25 ± 8.62 0.197	24.06 ± 8.36 28.33 ± 6.26 28.78 ± 9.66 0.218	0.027 *^a^ 0.655 0.374
MUIS					
	G1 G2 G3 *p*-value	48.95 ± 12.37 50.00 ± 9.20 49.61 ± 11.34 0.951	41.10 ± 12.11 46.25 ± 10.55 46.20 ± 11.04 0.259	35.66 ± 10.62 40.05 ± 12.40 44.00 ± 12.47 0.180	0.006*^a^ <0.001*^a^ 0.353
CSI					
	G1 G2 G3 *p*-value	35.15 ± 20.91 35.20 ± 13.47 27.09 ± 11.45 0.156	30.55 ± 20.82 32.63 ± 12.42 22.42 ± 11.35 0.106	24.06 ± 17.61 30.23 ± 12.53 21.42 ± 13.67 0.237	0.284 0.477 0.316
EQOL-5D					
	G1 G2 G3 *p*-value	7.65 ± 3.89 6.08 ± 2.88 6.33 ± 3.10 0.261	5.15 ± 3.89 4.60 ± 3.15 4.50 ± 2.92 0.805	4.73 ± 3.75 4.66 ± 3.37 5.21 ± 3.59 0.901	0.053 0.212 0.187

* *p* < 0.05; ^a^ post hoc: difference between baseline and day 45; Abbreviations: PCS, Pain Catastrophizing Scale; TSK, Tampa Scale of Kinesiophobia; MUIS, Mishel Uncertainty in Illness Scale; CSI, Central Sensitization Index; EQOL, Euro Quality of Life.

**Table 4:** Treatment effects on physical function variables at specified time points (days from baseline). All values are means and standard deviation.

Outcome		T1 (D1) G1 = 20 G2 = 24 G3 = 21	T2 (D15) G1 = 20 G2 = 20 G3 = 20	T4 (D45) G1 = 15 G2 = 18 G3 = 14	*p*-Value
6-Minute Walk Test					
	G1 G2 G3 *p*-value	238.84 ± 66.33 242.46 ± 56.01 234.95 ± 49.87 0.909	255.70 ± 72.14 273.16 ± 51.14 247.75 ± 54.83 0.411	264.43 ± 64.71 283.06 ± 43.59 258.23 ± 57.88 0.423	0.544 0.031 *^a^ 0.460
10-Meter Walk Test					
	G1 G2 G3 *p*-value	18.58 ± 6.43 17.65 ± 4.92 18.80 ± 5.54 0.763	17.17 ± 6.70 15.53 ± 3.31 17.65 ± 5.01 0.409	16.03 ± 4.66 14.46 ± 2.53 16.93 ± 6.34 0.316	0.471 0.029 *^a^ 0.604
SPPB					
	G1 G2 G3 *p*-value	7.20 ± 2.54 7.08 ± 2.18 7.19 ± 2.29 0.983	8.55 ± 2.03 8.25 ± 1.99 8.30 ± 1.75 0.871	8.53 ± 2.09 8.61 ± 2.11 8.35 ± 2.37 0.947	0.114 0.052 0.170
Force of Affected Right Leg (n = 32)					
	G1 G2 G3 *p*-value	12.31 ± 3.68 11.37 ± 3.61 9.55 ± 3.85 0.240	13.63 ± 4.87 14.35 ± 4.90 11.67 ± 3.34 0.384	15.15 ± 5.59 13.81 ± 4.74 11.36 ± 1.93 0.245	0.475 0.296 0.286
Force of Affected Left Leg (n = 33)					
	G1 G2 G3 *p*-value	11.52 ± 2.12 9.49 ± 2.63 13.84 ± 5.26 0.023 *	13.90 ± 3.56 12.90 ± 4.39 14.43 ± 5.17 0.719	13.96 ± 2.65 14.53 ± 4.00 14.08 ± 5.32 0.950	0.123 0.008 *^a^ 0.970

* *p* < 0.05; ^a^ post hoc: difference between baseline and day 45. Abbreviations: D, day; SPPB; Short Physical Performance Battery seconds.

**Table 5:** *p*-Values of statistical significance for differences across groups (G1 vs. G2 vs. G3) after multiple imputation of missing data for the treatment effects on pain at specified time points (days from baseline).

Outcome	T3 (D30)	T4 (D45)	T5 (D180)
VAS	0.663	0.675	0.583
WOMAC (total)	0.503	0.814	0.586
WOMAC (pain)	0.949	0.882	0.213
WOMAC (stiffness)	0.237	0.928	0.919
WOMAC (function)	0.664	0.838	0.410

Abbreviations: VAS, visual analogue scale; WOMAC, Western Ontario McMaster Universities Osteoarthritis Index; D, day.

## Data Availability

Data are contained within the article.
